# Supplementary figures and images for: The impact of genetically controlled splicing on exon inclusion and protein structure
Source: PLoS One. 2024 Mar 13;19(3):e0291960. doi: 10.1371/journal.pone.0291960 (PMC10936842; doi:10.1371/journal.pone.0291960)

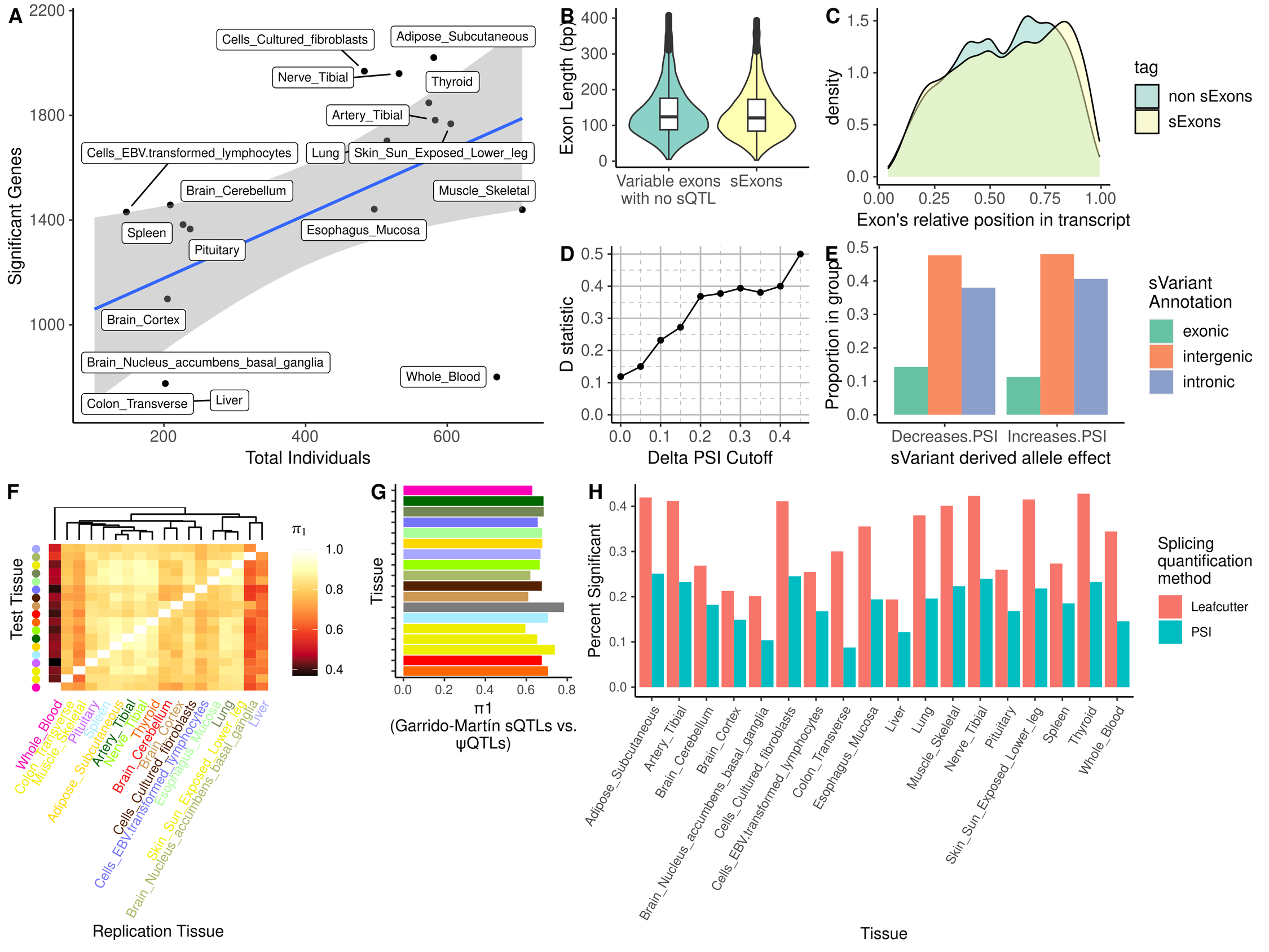

Supplement: S1 Fig — A) Relationship between the number of individuals tested and the number of significant ψQTLs per tissue. We catalog more significant genes in tissues where more donors are available, as is typical in QTL studies. B) Distribution of lengths between variable exons with and without a significant sVariant controlling splicing levels. While statistically significant, the difference is not large. C) Density plot of variable exons’ relative position in their respective transcripts. Across both groups, variable exons tend to occur later in the transcript. D) Increase in difference between derived allele frequency distributions when increasing the ψQTL effect size cutoff. The Kolmogorov-Smirnov D score, which quantifies the degree of difference between two distributions, increases as we consider stronger ψQTLs. E) Annotations of top sVariants across all ψQTL genes, split by derived allele effect direction on PSI. F) ψQTL replication heatmap between GTEx tissues. For a test tissue, we extract the nominal p-value of all significant (beta-corrected p < .05) variant-exon pairs from the 17 other test tissues, where data is available. From these sets of p-values, we calculate Storey’s π1 value, which represents the estimated fraction of true positive ψQTLs that were first discovered in the test tissue. The y axis is labeled with the same color key as the x axis. G) π1 scores, calculated by retrieving the p-value from all significant sQTLs in Garrido-Martín et al. [18] and testing the same variant in the ψQTLs. H.) Percentages of tested genes with a significant ψQTL across tissues, compared to genes with an sQTL with splicing mapped using Leafcutter. (TIF) [file pone.0291960.s001.tif]

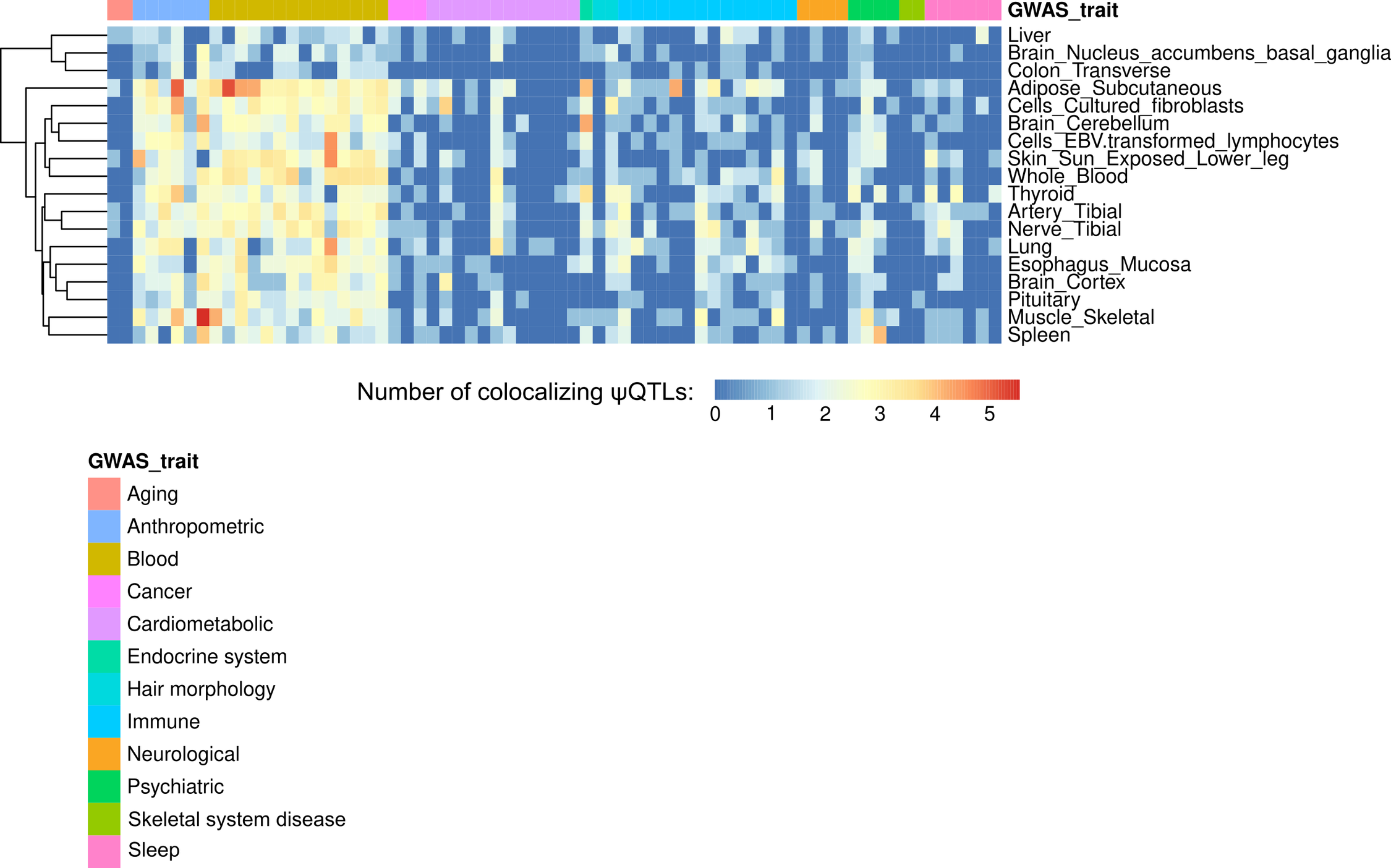

Supplement: S2 Fig — Counts of ψQTL-GWAS colocalization events across the 18 tested tissues. Traits are colored by their broad category, and rows are organized by hierarchical clustering. (TIF) [file pone.0291960.s002.tif]

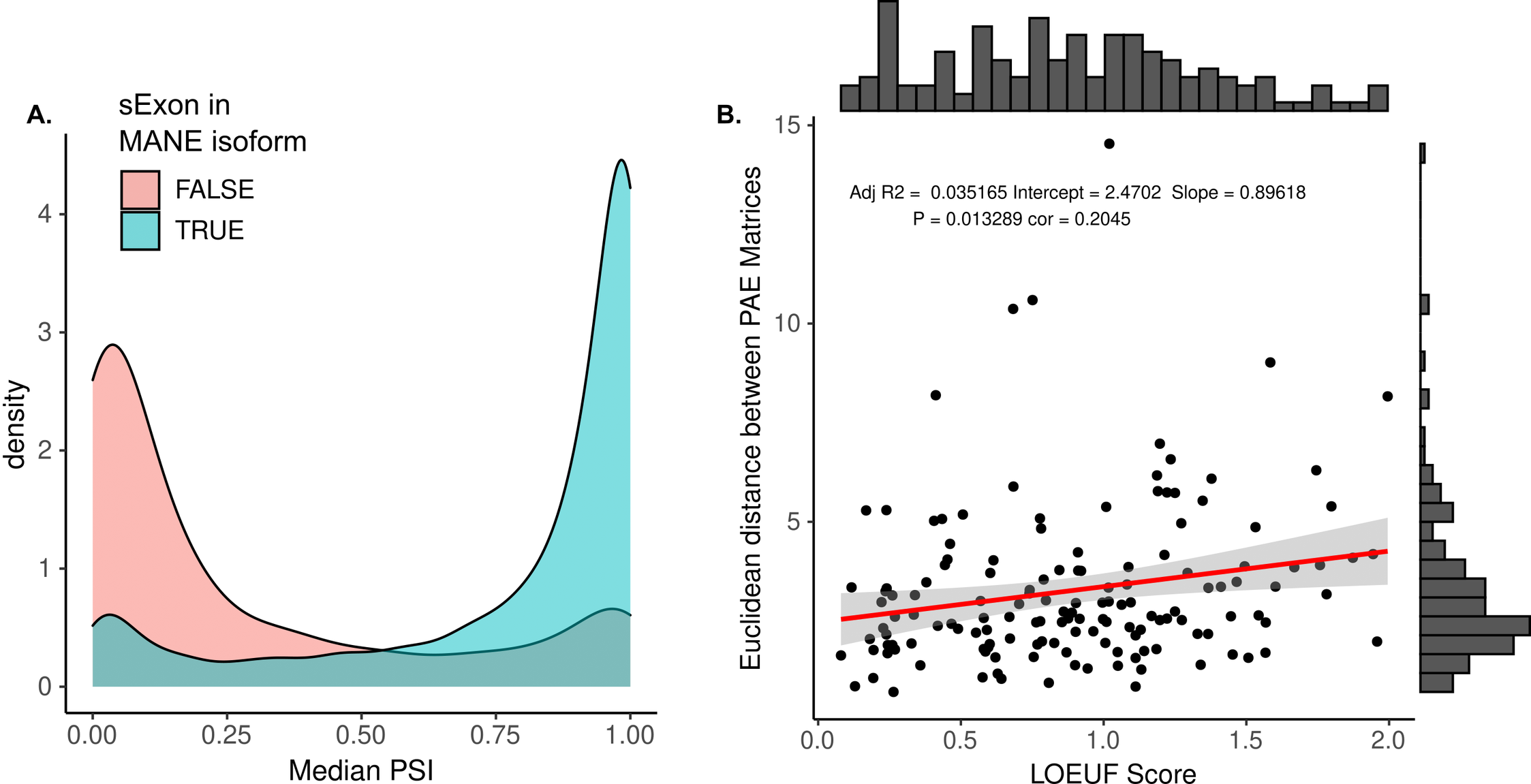

Supplement: S3 Fig — A) Distribution of median PSI scores between exons included or excluded in their respective gene’s MANE isoform. B) Genes with larger structural changes between trait-associated isoforms are also less likely to be haploinsufficient. This suggests genes which tolerate regulatory variants with large splicing effect sizes are also more tolerant to loss-of-function coding variants. (TIF) [file pone.0291960.s003.tif]

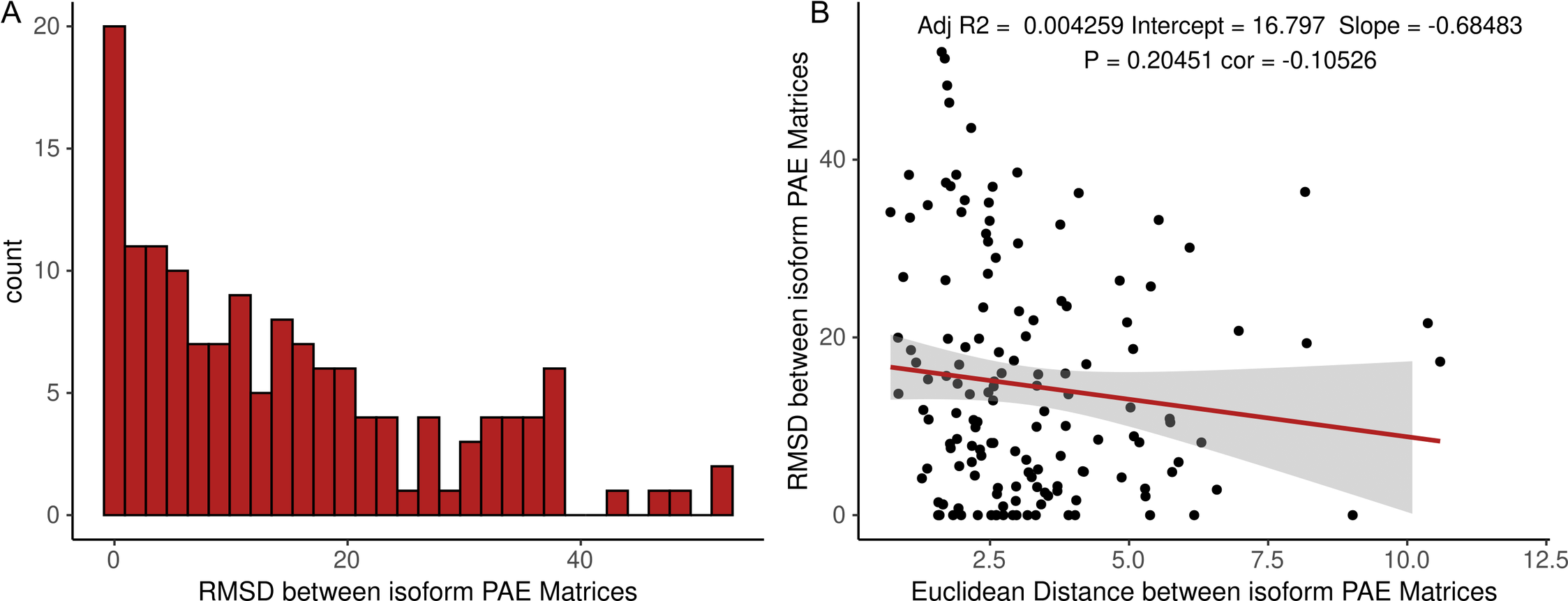

Supplement: S4 Fig — A) Distribution of Root Mean Squared Distance (RMSD) between spliced in and spliced out ψQTL isoforms that colocalize with a GWAS trait. B) Correlation between RMSD and the Euclidean Distance between isoform PAE matrices. These two quantities are not significantly correlated. (TIF) [file pone.0291960.s004.tif]
