# Supplementary material for: The impact of genetically controlled splicing on exon inclusion and protein structure
Source: PLoS One. 2024 Mar 13;19(3):e0291960. doi: 10.1371/journal.pone.0291960 (PMC10936842; doi:10.1371/journal.pone.0291960)
Supplement: S1 Table — (DOCX) [file pone.0291960.s005.docx]

Table S1: Total number of exons with PSI covered across tissues

| Tissue | N Exons per tissue pre-filtering | N Exons per tissue post-filtering | Percent usable | Genes covered per tissue |
| --- | --- | --- | --- | --- |
| Adipose_Subcutaneous | 260,800 | 29,180 | 11.19% | 8,585 |
| Artery_Tibial | 253,109 | 27,453 | 10.85% | 8,127 |
| Brain_Cerebellum | 239,928 | 36,095 | 15.04% | 8,605 |
| Brain_Cortex | 240,439 | 26,121 | 10.86% | 7,857 |
| Brain_Nucleus_accumbens_basal_ganglia | 247,074 | 26,372 | 10.67% | 7,998 |
| Cells_Cultured_fibroblasts | 230,752 | 28,486 | 12.34% | 8,479 |
| Cells_EBV.transformed_lymphocytes | 220,547 | 37,837 | 17.16% | 9,291 |
| Colon_Transverse | 231,647 | 29,066 | 12.55% | 8,630 |
| Esophagus_Mucosa | 245,627 | 26,721 | 10.88% | 7,984 |
| Liver | 224,469 | 21,605 | 9.62% | 6,283 |
| Lung | 265,555 | 34,585 | 13.02% | 9,387 |
| Muscle_Skeletal | 240,921 | 22,664 | 9.41% | 6,788 |
| Nerve_Tibial | 261,375 | 30,771 | 11.77% | 8,783 |
| Pituitary | 259,310 | 32,795 | 12.65% | 8,774 |
| Skin_Sun_Exposed_Lower_leg | 259,438 | 29,570 | 11.40% | 8,588 |
| Spleen | 241,122 | 30,379 | 12.60% | 8,277 |
| Thyroid | 266,364 | 30,035 | 11.28% | 8,586 |
| Whole_Blood | 236,866 | 23,135 | 9.77% | 6,039 |
