# Supplementary material for: The impact of genetically controlled splicing on exon inclusion and protein structure
Source: PLoS One. 2024 Mar 13;19(3):e0291960. doi: 10.1371/journal.pone.0291960 (PMC10936842; doi:10.1371/journal.pone.0291960)
Supplement: Attachment — Submitted filename: Response to Reviewers - 2.13.23.docx [file pone.0291960.s006.docx]

Dear Dr. Liu,

Thank you for reviewing our manuscript titled *The Impact of genetically controlled splicing on exon inclusion and protein structure.* We are pleased to address your comments. Our revisions are as follows:

1) We have updated the manuscript to meet the PLOS ONE style requirements, both in the body text and the author affiliations.
2) We have updated the financial disclosure and funding information section to match. We were unable to include the S. Jay Levy Foundation or the Swedish National Infrastructure for Computing on the online portal, as these did not appear to be listed as known funding sources in the database.
3) We have removed all funding information from the acknowledgement section. See following point for our finalized Funding Statement, to be added to the online submission.

Updated Funding Statement:

This work was supported by the National Institutes of Health grants R01GM122924, R01MH106842 (J.E. & T.L.), the Data Driven Life Science program by the Knut and Alice Wallenberg Foundation grant WASPDDLS21:080 (M.M. & T.L.) and the S. Jay Levy Foundation (https://www.ccny.cuny.edu/sjaylevy) (F.R.). Part of the computations were enabled by resources provided by the Swedish National Infrastructure for Computing (SNIC) at UPPMAX partially funded by the Swedish Research Council through grant agreement no. 2018-05973. (M.M. & T.L.)

4) We confirm that our stated conflicts of interest do not alter our adherence to PLOS ONE’s policy on data sharing. Please include the following updated statement:

Updated Competing Interests Statement:

I have read the journal's policy and the authors of this manuscript have the following competing interests: T.L. is a paid advisor to GSK, Pfizer, Goldfinch Bio and Variant Bio, and has equity in Variant Bio. This does not alter our adherence to PLOS ONE policies on sharing data and materials.

5) Please include the following data availability statement.

Updated Data Availability Statement:

Raw and processed data files are available to download at https://zenodo.org/record/7275062 and at gtexportal.org. Individual level genetic data is necessary for QTL mapping, but cannot be shared publicly because of NIH policy, since it contains potentially sensitive information that could be used to identify anonymous GTEx donors. You must request access via dbGAP if you require these data (https://www.ncbi.nlm.nih.gov/projects/gap/cgi-bin/study.cgi?study_id=phs000424.v8.p2). Protected data are available in the AnVIL repository.

Accompanying code is available to review and download at https://github.com/jeinson/sqtl_manuscript

In response to additional comments, the following revisions were made to the manuscript.

1) We performed ψQTL calling using RNA sequencing and genotyping from the Geuvadis project, and compared the number of sGenes discovered with both methods. We observed high concordance, which builds evidence that these results are replicable across multiple datasets. Results and methods from this analysis are described in the body text.
2) To investigate the robustness of ψQTLs across tissue, we performed concordance analysis of our significant variant-gene pairs across using Storey’s π_1_ method, and found good replication of our results. This analysis is reported in the text, and we included an additional panel added to Supplemental Figure 1 (Fig S1G).

We hope with these additional changes, you can distribute our article for peer review.

Thank You,

Jonah Einson
